# Heterogeneous deposition of regular and mentholated little cigar smoke in the lungs of Sprague-Dawley rats

**DOI:** 10.1186/s12989-023-00554-6

**Published:** 2023-11-06

**Authors:** Kaisen Lin, Christopher Wallis, Emily M. Wong, Patricia Edwards, Austin Cole, Laura Van Winkle, Anthony S. Wexler

**Affiliations:** 1 Department of Civil and Environmental Engineering, Michigan State University, East Lansing, MI 48824 USA; 2 Air Quality Research Center, University of California, Davis, Davis, CA 95616 USA; 3 Department of Anatomy, Physiology and Cell Biology, University of California, Davis, Davis, CA 95616 USA; 4 Center for Health and the Environment, University of California, Davis, Davis, CA 95616 USA; 5 UC Davis Interdisciplinary Center for Plasma Mass Spectrometry, University of California, Davis, Davis, CA 95616 USA; 6 Department of Mechanical and Aerospace Engineering, University of California, Davis, Davis, CA 95616 USA; 7 Department of Civil and Environmental Engineering, University of California, Davis, Davis, CA 95616 USA; 8 Department of Land, Air, and Water Resources, University of California, Davis, Davis, CA 95616 USA

**Keywords:** Little cigar smoke particles, Lung lobar deposition, Sprague-Dawley rats, Heterogeneous deposition, MPPD model

## Abstract

**Background:**

Quantifying the dose and distribution of tobacco smoke in the respiratory system is critical for understanding its toxicity, addiction potential, and health impacts. Epidemiologic studies indicate that the incidence of lung tumors varies across different lung regions, suggesting there may be a heterogeneous deposition of smoke particles leading to greater health risks in specific regions. Despite this, few studies have examined the lobar spatial distribution of inhaled particles from tobacco smoke. This gap in knowledge, coupled with the growing popularity of little cigars among youth, underscores the need for additional research with little cigars.

**Results:**

In our study, we analyzed the lobar deposition in rat lungs of smoke particles from combusted regular and mentholated Swisher Sweets little cigars. Twelve-week-old male and female Sprague-Dawley rats were exposed to smoke particles at a concentration of 84 ± 5 mg/m^3^ for 2 h, after which individual lung lobes were examined. We utilized Inductively Coupled Plasma Mass Spectrometry to quantify lobar chromium concentrations, serving as a smoke particle tracer. Our findings demonstrated an overall higher particle deposition from regular little cigars than from the mentholated ones. Higher particle deposition fraction was observed in the left and caudal lobes than other lobes. We also observed sex-based differences in the normalized deposition fractions among lobes. Animal study results were compared with the multi-path particle dosimetry (MPPD) model predictions, which showed that the model overestimated particle deposition in certain lung regions.

**Conclusions:**

Our findings revealed that the particle deposition varied between different little cigar products. The results demonstrated a heterogenous deposition pattern, with higher particle deposition observed in the left and caudal lobes, especially with the mentholated little cigars. Additionally, we identified disparities between our measurements and the MPPD model. This discrepancy highlights the need to enhance the accuracy of models before extrapolating animal study results to human lung deposition. Overall, our study provides valuable insights for estimating the dose of little cigars during smoking for toxicity research.

**Supplementary Information:**

The online version contains supplementary material available at 10.1186/s12989-023-00554-6.

## Introduction

Inhalation exposure to particulate matter from many sources can lead to adverse health effects and result in respiratory and cardiovascular disease and even death [1–3]. For tobacco smoke, quantifying the dose and identifying location of deposited particles in the respiratory tract are essential for understanding their toxicity, addiction potential, and overall health effects. The pattern of particle deposition in the respiratory tract depends on factors such as particle size [4, 5], chemical composition of inhaled particles [6, 7], breathing pattern of individuals [8, 9], as well as the physical characteristics of the airway [10]. For example, both animal study results and model predictions suggest that larger particles primarily deposit in the upper respiratory tract, while smaller particles tend to penetrate deeper into the lungs, settling in the alveolar region [11]. Airway geometry and breathing pattern, which can vary substantially with age, body size, and health condition of subjects, also greatly influence particle deposition patterns [12, 13].

These factors not only impact the overall lung particle dose, but also influence the distribution of particles at a finer resolution, such as lobe-by-lobe or airway generation-by-generation. Heterogeneous particle deposition in the lungs presents a significant health risk especially to 'hotspot' regions, where the smoke dose is higher, potentially leading to an increased risk of disease development. Previous research discovered that 69% of tumors in cigarette smokers were located in the peripheral airway, compared to 31% in the central airway, indicating disparities in tumor development across different lung structures [14]. In contrast, other research reported homogeneous deposition and even retention across the lobes of Sprague-Dawley rat when exposed to much smaller particles that have distinct hygroscopic growth and evaporation characteristics, such as 20 nm silver nanoparticles [15]. These seemingly contradictory results from different studies highlight the complexity of particle deposition in the respiratory tract and reinforces the need for further investigation into spatial deposition patterns (e.g., lobe-specific deposition fraction or hotspots of particle deposition) with different types of particles and animal models. Findings from such investigations inform toxicity studies to identify locations where concerning health risks may arise, offering a more comprehensive understanding of the adverse health effects from particle exposure. However, few research efforts have explored the spatial distribution of particles from inhalation exposure.

Tobacco smoking contributes to over seven million deaths per year, a leading cause of death worldwide, and can lead to a variety of lung, heart, and pulmonary diseases [1, 16]. In 2022, the FDA proposed to ban menthol as a flavor in cigarettes and all other flavors in cigars [17]. Yet, little cigars, which serve as alternatives and are unregulated for flavors, are gaining popularity among minority groups and adolescents [18]. Unlike cigarettes, little cigars are wrapped in a tobacco leaf and offer a variety of flavors, including menthol and fruits. These flavors, perceived as less harsh, enhance their addictive potential [19]. Despite being seen as less harmful than cigarettes by young adults [20], studies have shown that little cigars may be even more toxic [21, 22]. Their accessibility, due to small packages and affordability compared to other tobacco products, also makes them appealing to adolescents [23]. However, to our knowledge, no studies have been conducted on the deposition of little cigar smoke in lungs, leaving a significant gap in our understanding of the dose and the health outcomes of these harmful products.

Mathematical models, developed over past decades, have been used to predict particle deposition [24–26]. In theory, they could be useful for predicting the deposition patterns of little cigar smoke particles in animal or human lungs. However, due to uncertainties from various input parameters, it is extremely challenging to predict accurately how much and where particles will deposit in subjects' lungs. Additionally, since lobe-specific particle deposition measurement data are limited, models often perform poorly in estimating particle deposition across lobes. Collecting data from animal model experiments will be invaluable for improving model performance, which can then be used to extrapolate results from the rat model to the human lung.

This study is motivated by the rising popularity of little cigars and a noticeable gap in existing literature concerning the lung deposition of particles resulting from their combustion. We focused on the lobe-specific deposition patterns of regular and mentholated little cigar smoke particles in the lungs of Sprague-Dawley rats and juxtaposed our findings with model predictions. The objectives of this research were twofold: to explore the deposition pattern of little cigar smoke and to evaluate the performance of the Multiple Path Particle Dosimetry (MPPD) model in predicting the deposition of little cigar smoke particles.

## Materials and methods

### Animals and their care

Eleven-week-old male and female Sprague-Dawley rats, purchased from Envigo (Livermore, CA), were housed in pairs (males) and trios (females) in a vivarium with controlled temperature (72 °F) and relative humidity (20–60% depending on seasons), adhering to a 12-h light–dark cycle. Rodent diet and water were provided ad libitum. Rats acclimated for one-week prior to study exposures. The mean body weights of the males and females were 332 ± 11 and 221 ± 13 g, respectively, on the first day of exposure. All experiments were carried out at the Center for Health and the Environment at the University of California, Davis. The study adhered to federal guidelines for the use and care of laboratory animals, and the experimental protocols were reviewed and approved by the Institutional Animal Care and Use Committee at the University of California, Davis.

### Design of experiment

Four exposure experiments were conducted, each encompassing one sex (male or female) exposed to one type of little cigar smoke (regular or mentholated). For each experiment, fifteen rats were randomly divided into three groups of five each: the exposure group (EG), the control group (CG), and the blank group (BG). Rat tails were color-marked for identification prior to exposure. The rats in both the EG and CG were exposed to little cigar smoke on Days 1, 2, and 3. The aim of the initial three-day exposure was to acclimate the rats, allowing them to adapt their breathing patterns upon smoke exposure to yield representative results. Exposure was halted on Day 4 to lower baseline particle levels in lung tissues. On Day 5, only the rats in the EG were exposed, while the CG remained unexposed. By subtracting the CG group from the EG group, any particle dissolution and/or translocation that occurred prior to the exposure on Day 5 is accounted for and removed. The BG was never exposed to little cigar smoke, though they were brought to the same room on exposure days and placed in an empty fume hood for sham exposure.

### Exposure experiment

**Fig. 1:**
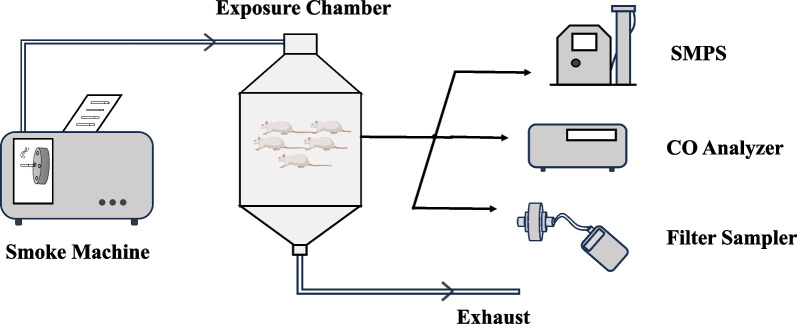
Schematic of the exposure experiment setup. Little cigar smoke particles were generated inside the smoke machine, which were then directed and introduced to the exposure chamber. Particle size distribution and CO level were monitored by Scanning Mobility Particle Sizer (SMPS) and a CO analyzer. Smoke particulate was collected on a 25 mm PTFE filter by a SKC pump at 1 L per minute every five minutes for further analysis

The animal exposure experiments were conducted using the setup depicted in Fig. 1. The customized smoke machine, designed for loading, lighting, and puffing Swisher Sweets regular and mentholated little cigars (Swisher Sweets), consists of a little cigar magazine, a piston, a 10-spot rotating wheel, a heating coil lighter, a diaphragm puff pump, and a control module connected to a tablet via Bluetooth. Detail information on the configuration and operation of this smoke machine were reported previously with minor modifications [27]. The machine loads a batch of 10 little cigars onto the rotating wheel, which rotates every 6 s, lighting and subsequently puffing each little cigar once every minute. Cooperation Centre for Scientific Research Relative to Tobacco’s method 64 Routine Analytical Cigar-smoking Machine Specification, Definition, and Standard Conditions (20 mL puff volume and 1.5 s puff duration) was initially utilized as the puffing regimen. However, little cigars did not remain lit using this puffing standard. To address this issue, a 50 mL, 3-s puff with a 60-s interval puffing regimen was followed, which was derived from the FDA's FTC/ISO and the Massachusetts Department of Public Health Protocol. All little cigars lasted approximately 10 min before they burned completely and were discarded. A new batch was loaded and lit immediately afterward. The resultant mixture of mainstream and sidestream smoke was directed to the animal exposure chamber at a rate of 20 L per minute, with the air flow rate measured by a TSI 4046 flow calibrator.

The animal exposure facility includes three serial stainless steel whole-body exposure chambers, each with a volume of 440 L. During the exposure experiment, the ambient temperature ranged between 21 and 24 °C, while the relative humidity varied between 55 and 70%. The chambers were designed such that air entered from the top and exited from the bottom. As there was a minor decrease in particle mass concentration across the chambers, exposure took place solely in the first chamber to maintain consistent exposure concentration. Each exposure lasted 2 h. Rats were transferred to clean cages and returned to the vivarium after each exposure. Immediately after the exposure on Day 5, the body weight of the rats was measured and all rats from the three groups were subsequently euthanized.

### Particle samples and lung tissues collection

The exposure chamber is equipped with multiple sampling ports, enabling continuous monitoring of particle size distribution, particle mass concentration, and carbon monoxide (CO) concentration. Particle sizes in the range of 14–661 nm were characterized using a Scanning Mobility Particle Sizer (SMPS, TSI). Gravimetric analyses of 25 mm PTFE filters (Pall Corporation), conducted before and after particle collection, were used to estimate the particle mass concentration to which the rats were exposed. Weighing of the air filters, which were equilibrated to 40% RH in a desiccator for at least 24 h, was carried out with a microbalance (XP26, Mettler Toledo) and anti-static kit (EN-C, Mettler Toledo). Smoke particles were recovered from these air filters and quantified for metal concentrations by Inductively Coupled Plasma Mass Spectrometry (ICP-MS). CO levels were monitored by an X-Stream Gas Analyzer (Rosemount Analytical).

Lung tissues were collected and separated following a protocol similar to a previously reported method [28]. After the final day’s exposure experiment, each rat was euthanized intraperitoneally with 5 mL Fatal-Plus solution per kg of body weight and mounted on a surgical board. The lungs and extrapulmonary airways were removed *en bloc*, with the trachea cut below the larynx. Lobes were carefully separated at the lobar bronchus into left, accessory, cranial, caudal, and medial lobes. Each lobe was immediately placed in 2 mL microcentrifuge tubes and flash-frozen in liquid nitrogen to prevent particle translocation. These lobe samples were then stored at − 80 °C prior to digestion for ICP-MS analysis. The necropsy and lung sample collection process, i.e. for 15 rats, was typically completed within 1 h of exposure.

### Sample preparation for ICP-MS analysis

Lung tissues were subsequently acid-digested and analyzed with ICP-MS at the Interdisciplinary Center for Plasma Mass Spectrometry at the University of California, Davis. The digestion process involved adding 1 mL of 50% nitric acid (HNO_3_) and 334 µL of concentrated trace metals grade hydrogen chloride (HCl), followed by a 10-min incubation at room temperature. The samples were then heated in a dry bath at 95 °C for 10 min. Subsequently, 500 µL of concentrated HNO_3_ was added, and the samples were heated at 95 °C for 40 min. Following a cool down period, 1.5 mL of 30% hydrogen peroxide (H_2_O_2_) was incrementally added to the samples and the samples were heated at 95 °C for 2 h. After cooling, samples were centrifuged and brought up to a final volume of 3 mL with Milli-Q water. For samples weighing more than 0.3 g, double the reagents were added to ensure sufficient digestion.

Particle-loaded air filters were processed following a previously described method [29]. Filters were transferred to 50 mL vials containing a 10 mL mixture of 75% acetone and 25% 1N HNO_3_, and ultrasonicated for 30 min to recover particles. Filters were weighed before and after extraction to determine extraction efficiency. This method effectively and consistently recovered more than 85% of the captured particles (Additional file 1: Table S1). The 50 mL vials containing the extracts were placed on a dry bath at 95 °C to evaporate the acetone. Smoke particle extracts were then concentrated to approximately 100 µL and digested with 250 µL of concentrated HNO_3_ at 95 °C for 2 h. The digested solutions were brought up to a final volume of 2.5 mL with Milli-Q water before ICP-MS analysis.

Lung digests and filter extracts were analyzed by Agilent 8900 ICP-MS Triple Quad instrument (Agilent Technologies) equipped with an inert PFA sample introduction kit, Pt cones, and a brass baseplate. ICP-MS instrument and detailed operation conditions can be found in the Additional file 1.

### Deposition calculation

Metals, naturally present in tobacco and subsequently in smoke particles due to the combustion of little cigars, were used to trace the deposition of particles in the lungs of Sprague-Dawley rats. As these metals were inhaled and deposited along with tobacco smoke particles, their deposition patterns in rat lungs can accurately represent those of smoke particles.

The deposition fraction was calculated as the ratio of the mass of deposited metals to the mass of inhaled metals, as shown in Eq. 1. Specifically, the mass of deposited metals was the difference in metal quantities between rats in EG and CG. The impacts of metal dissolution and clearance in EG rats prior to Day 5’s exposure, if there were any, will be effectively eliminated using this method. Since lung tissues were collected immediately, metal dissolution and clearance occurred post-exposure on Day 5 should have minimal impacts on the results. The mass of inhaled metals was determined by the product of metal concentration in smoke, the minute volume of rats, and exposure duration (Eq. 2). The minute volume was determined to be 0.26 and 0.20 L per minute for males and females, respectively, by the MPPD model based on mean body weight. To account for variations in lobe size, deposition fraction was normalized with lobe-specific airway surface areas, yielding the surface area-normalized deposition fraction (Eq. 3).

1 $$Deposition\,fraction= \frac{Mass\,of\,deposited\,metals}{Mass\,of\,inhaled\,metals}$$

2 $$Mass\,of\,inhaled\,metals = Metal\,conc.\,in\,air\,\times\,Rat\,minute\,volume\,\times\,Exposure\,duration$$

3 $$Normalized\,deposition\,fraction = \frac{Deposition\,fraction}{Lobe\,specific\,airway\,surface\,areas}$$

### Model prediction with MPPD

Particle deposition in rat lungs was modeled using the publicly available MPPD model (EPA 2020 Beta v1.01). The model's outcomes were compared with the results of the exposure experiment. To ensure accurate comparisons, modeling parameters were set to match those used in the animal study. These parameters included animal model species, body weight, airway characteristics, exposure scenario, and characteristics of smoke particles. Certain parameters were determined based on existing literature. For instance, smoke particle density was set at 1.180 g/cm^3^ as reported by previous studies on reference cigarette mainstream smoke [30], which is assumed to be representative for the mixed mainstream and sidestream smoke of little cigar. Remaining parameters were left at the model's default values based on animal mean body weights. Prior research showing that respiratory minute volume (RMV) decreases approximately 30% or less at 1000 ppm CO level in Sprague-Dawley rats [31, 32]. Given that our study consistently recorded CO levels below 500 ppm, a modest reduction in RMV was expected. Our study did not directly measure these breathing parameters, making it challenging to pinpoint the exact reduction percentage in RMV. However, due to the sensitivity of the MPPD model to changes in breathing parameters, we decided to use the default breathing frequency and tidal volume in the MPPD model, with the understanding that this will result in the model overestimating particle deposition. Table 1 summarizes input parameters for modeling male and female rats.

**Table 1:** Summary of multi-path particle dosimetry model input parameters

Input parameter	Male	Female
Species	Rat	Rat
Model	Asymmetric Sprague-Dawley	Asymmetric Sprague-Dawley
Body weight (gram)	332	221
FRC volume (mL)	3.69	3.02
URT volume	0.44	0.34
Particle density (g/cm^3^)	1.180	1.180
Aspect ratio	1	1
Diameter (CMD) (nm)	359	359
Geometric SD	1.79	1.79
Concentration (mg/m^3^)	84	84
Breathing frequency (/minute)	113	125
Tidal volume (mL)	2.32	1.62
Inspiratory fraction	0.5	0.5
Pause fraction	0	0
Breathing scenario	Whole body exposure	Whole body exposure
Dose metric	Deposition only	Deposition only

### Statistical analysis

The statistical analysis was conducted using RStudio. Data on lobe-specific deposition fractions and normalized deposition fractions within the same sex, i.e., male or female rats, were analyzed using One-way ANOVA, with a post-hoc TukeyHSD test applied to identify significant differences. The model predictions and results from the animal study were tested for statistical significance using the Kruskal–Wallis test, with a post-hoc Dunn’s test. *P*-values less than 0.05 were considered statistically significant.

## Results

### Smoke particle characteristics

**Fig. 2:**
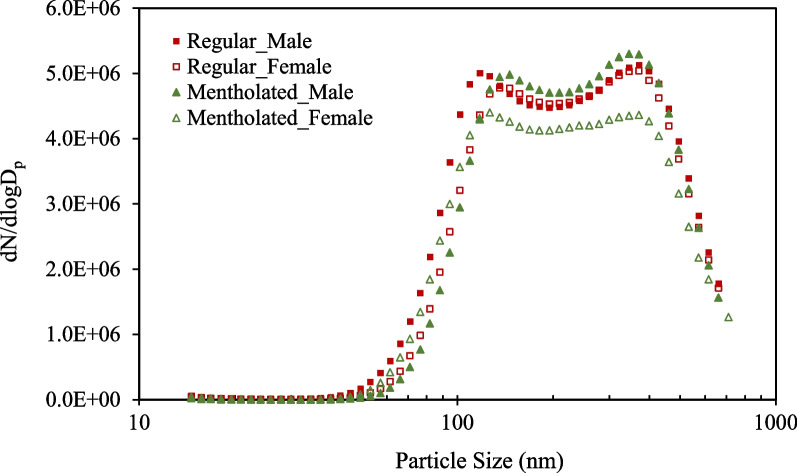
Averaged particle size distributions of regular and mentholated little cigar smoke measured by SMPS. For each flavor, exposure experiments were conducted separately for male and female rats due to the limited capacity of the exposure chamber. Each dot represents the average particle number concentration at a given particle size over 2 h measurement. Standard deviation was not indicated in the figure to aid clear visualization of the curves

The size distribution of particles from little cigar smoke was determined using a TSI SMPS. This instrument performed continuous measurements at 2-min intervals throughout the 2 h exposure. By averaging the particle concentration for each particle size over time, the average size distribution for each exposure was determined. Despite minor variations in particle size and mass concentration, the exposure scenarios across the four experiments showed a high level of consistency. As shown in Fig. 2, bimodal distributions were observed during the exposure experiment. The little cigar smoke displayed a primary peak at 359–372 nm, likely resulting from rapid coagulation of smaller particles due to the high number concentration. A secondary peak was identified at 122–146 nm. The observed variations in particle size across exposure experiment are subtle enough to not affect the spatial distribution of smoke particles in different lobes of rats.

The flavor of little cigar products did not significantly affect the particle mass concentration. The particle mass concentrations over the 2-h exposure period showed minor variations among different experiments, with an average of 84 ± 5 mg/m^3^. The exposure concentration was higher than what might typically occur in reality. Nevertheless, due to the limit of quantification (LOQ) of ICP-MS analysis and the anticipated low particle deposition fraction, subjecting rats to a high exposure concentration was necessary to ensure reliable analysis results. The highest particle mass concentration was observed in the experiment where male rats were exposed to mentholated little cigars. In contrast, the lowest concentration was observed when female rats were exposed to the same type of little cigar. These differences in concentration are expected to have negligible effects on the total particle dose and should have no impact on the particle deposition fraction or normalized deposition fraction results. As for CO, its levels displayed an initial rapid increase in the first 30 min, and stabilized at 400–450 ppm for the remainder of each exposure.

This study was primarily centered on understanding the deposition patterns of the insoluble and soluble particulate constituents of smoke, setting aside the investigation of its volatile and semi-volatile components. A total of 31 elements in particle-loaded filter extracts and lung tissue digests were analyzed by ICP-MS. To identify the most suitable tracers of little cigar smoke particles, several criteria were applied. First, the levels of potential tracers in particle-loaded filter extracts should be significantly higher than those in extracts from blank filters. Second, the levels of these tracers in both filter extracts and lung tissue digests should exceed the LOQ, which is defined as three times the limit of detection (LoD). Third, the chosen tracers should have relatively low background abundances in the lung tissues of unexposed rats. Based on these criteria, chromium was identified as the most suitable tracer for little cigar smoke particles. It was found to be abundant in little cigar smoke with a concentration of 0.99 ± 0.11 ng/m^3^ and had a low abundance at 0.72 ppb (verses a LoQ of 0.20 ppb) in lung tissue prior to exposure. Hence, all subsequent calculations were based on chromium concentrations. As lung tissue samples were collected immediately post-exposure on Day 5, it is assumed that there was minimal Cr dissolution and translocation. Therefore, only total Cr was determined by ICP-MS, while Cr speciation was not further pursued.

### Deposition fraction

The deposition fraction of particles, i.e., the percentage of inhaled particles that were subsequently deposited, was calculated. The overall deposition fraction of regular little cigar smoke, representing the total of the left, accessory, caudal, cranial, and medial lobes, was 9.4% and 10.2% in male and female rat lungs, respectively. A notably lower overall deposition fraction was observed in the mentholated little cigar experiment, with values of 5.1% and 4.8% for males and females, respectively.

**Fig. 3:**
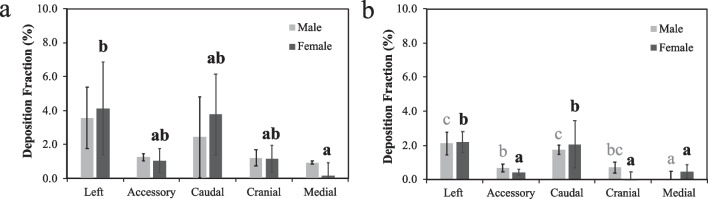
The lobe specific deposition fraction of **a** regular and **b** mentholated little cigar smoke in male and female rat’s lungs. Bars represent mean ± standard error for each lobe (n = 5). Bars with the different letters indicate significant difference (*P* < 0.05) between lobes within the same sex of rats according to one-way ANOVA with the Tukey’s HSD test. Statistical analyses on male and female rats’ data were conducted separately and thus the annotated letters should be interpreted separately

**Fig. 4:**
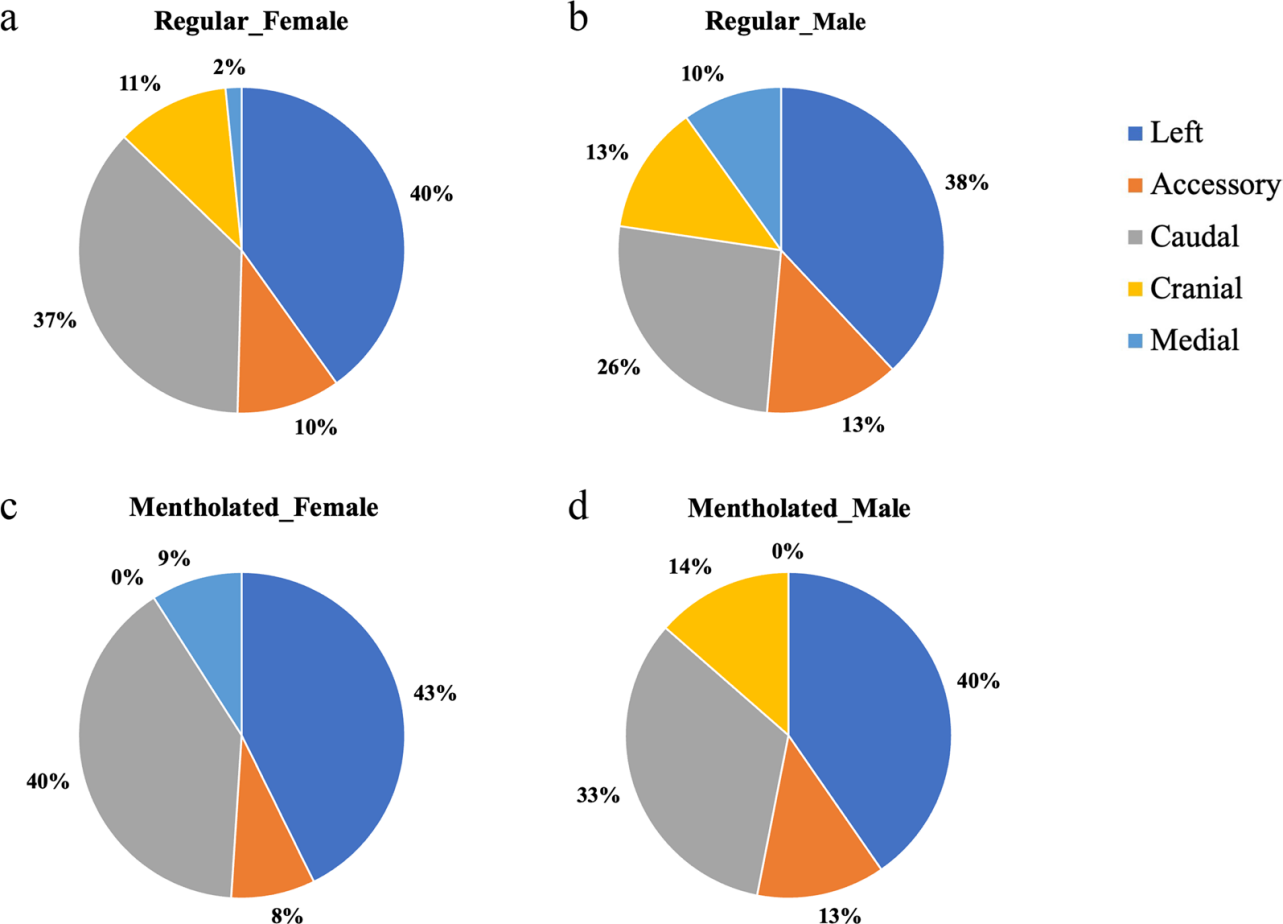
Percentage of particle deposition by lobe relative to total deposition for **a** female rats exposed to regular little cigar smoke, **b** male rats exposed to regular little cigar smoke, **c** female rats exposed to mentholated little cigar smoke, and **d** male rats exposed to mentholated little cigar smoke. For the pie chart, negative depositions observed in the cranial lobe of female rats and the medial lobe of male exposed to mentholated little cigar smoke were considered as zero

Lobe-specific deposition fractions (Fig. 3) were calculated to elucidate the spatial distribution of smoke particles in the rat lungs. A clear pattern of uneven particle deposition was observed for both types of little cigars and across both sexes: the left and caudal lobes consistently showed the highest deposition fraction among the five lobes. In the case of female rats exposed to regular little cigar smoke, their left lobes exhibited a significantly higher deposition fraction compared to their medial lobes. For male rats, accessory, cranial and medial lobes exhibited a low deposition of approximately 1%, although the difference with that in the left and caudal lobes were not statistically significant. The left and caudal lobes consistently accounted for over two-thirds of the total smoke deposited in the lung, whereas the medial lobe was responsible for less than 10% of the overall deposition (Fig. 4a, b).

Menthol, a flavor additive known for its cooling and analgesic effects [19], is added to tobacco products, making them more addictive. Due to the milder flavor, it is believed that smokers of mentholated products may inhale more deeply, retain the smoke longer, and thereby expose themselves to a higher amount of harmful chemicals [33]. A substantial reduction in both the overall and lobe-specific deposition fractions was observed during the mentholated little cigar experiment. This reduction in deposition fraction appeared to be consistent across all five lobes. Left and caudal lobes had significantly more deposition of mentholated little cigar smoke particles than the medial lobe for both sexes. A negligible deposition was observed in the cranial lobe of female rats and the medial lobe of male rats. In rats exposed to mentholated little cigar smoke, the left and caudal lobes contributed even more significantly to the total deposition, as illustrated in Fig. 4c, d.

### Normalized deposition fraction

The lobe-specific deposition fraction is valuable in estimating the total exposure and particle dose in each lobe. However, the results can be significantly influenced by lobe sizes. The left and caudal lobes typically possess larger volumes and greater airway surface areas compared to other lobes. To account for this, the deposition fraction was normalized by the airway surface areas of each lobe to calculate the normalized deposition fraction, which represents particle deposition fraction per unit area. Airway surface area data (Additional file 1: Table S2) were sourced from the MPPD model, as it allows for a fair comparison between model predictions and experimental results, a subject that will be elaborated upon in the following section.

**Fig. 5:**
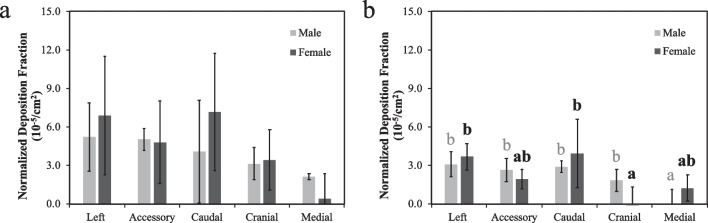
The lobe specific airway surface area-normalized deposition fraction of **a** regular and **b** mentholated little cigar smoke in male and female rat’s lungs. Bars represent mean ± standard error for each lobe (n = 5). Bars with the different letters indicate significant difference (*P* < 0.05) between lobes within the same sex of rats according to one-way ANOVA with the Tukey’s HSD test. Statistical analyses on male and female rats’ data were conducted separately and thus the annotated letters should be interpreted separately. There were no statistical differences in the normalized deposition fraction across the lobes for male and female rats exposed to regular little cigars. As a result, panel **a** does not contain any letter annotations

Similar to the deposition fraction, a heterogeneous pattern was observed among female rats, irrespective of the type of little cigars used (Fig. 5). A higher particle deposition per unit area was observed in the left and caudal lobes, which was significantly more than in the cranial lobe when the mentholated little cigar was tested. Conversely, a more uniform deposition pattern was observed in male rats. Both regular and mentholated little cigar smoke particles were deposited least in the medial lobe, with the other four lobes demonstrating similar normalized deposition fractions.

Female rats also displayed slightly higher normalized deposition fractions than male rats, particularly in the left and caudal lobes. This was primarily due to their smaller airway surface areas, which was about 88% of that in male rats. When comparing little cigars of different flavors, the regular little cigar resulted in a higher normalized deposition fraction than the mentholated cigars. Specifically, the overall lung normalized deposition fraction for female rats was 4.5 × 10^–5^/cm^2^ and 2.1 × 10^–5^/cm^2^ for regular and mentholated little cigars, respectively. Similar result was observed in male rats (3.9 × 10^–5^/cm^2^ and 1.9 × 10^–5^/cm^2^).

### Comparison of model prediction with measurements

The MPPD model is widely utilized to predict particle deposition in the respiratory tract of human and rodent models. It offers comprehensive and lobe-specific information on particle deposition, including deposition by airway generations and normalized deposition fraction by lobes. However, the chemical properties of particles can vary significantly and thus affect their deposition pattern in lungs, as evidenced by the difference observed between the regular and mentholated little cigar experiment. Different flavorings also have impacts on rats’ tidal volume, respiratory frequency, and the calculated minute volume during exposure. The aim of comparing model predictions with animal study results was to assess the accuracy of modeling outcomes and facilitate necessary model optimization.

One of the challenges in conducting a fair comparison between simulation and experiment was the observed bimodal distribution of little cigar smoke. The current version of the model does not accommodate the entry of the particle number concentration across the entire measured range. Informing the model with only a few data points can potentially oversimplify the exposure scenario and introduce errors in the model predictions. To examine the effect of particle size on lobe-specific deposition fractions, the model results for various particle sizes were compared. The model predicted that approximately 10% more particles would be deposited in rat lungs as particle size decreased from the primary peak at 359 nm to the secondary peak at 141 nm. This, however, did not influence the lobe-by-lobe deposition pattern, as the normalized deposition fraction in different lobes always increased or decreased to the same extent. Based on this, 359 nm was entered as the representative smoke particle size for subsequential model predictions.

**Fig. 6:**
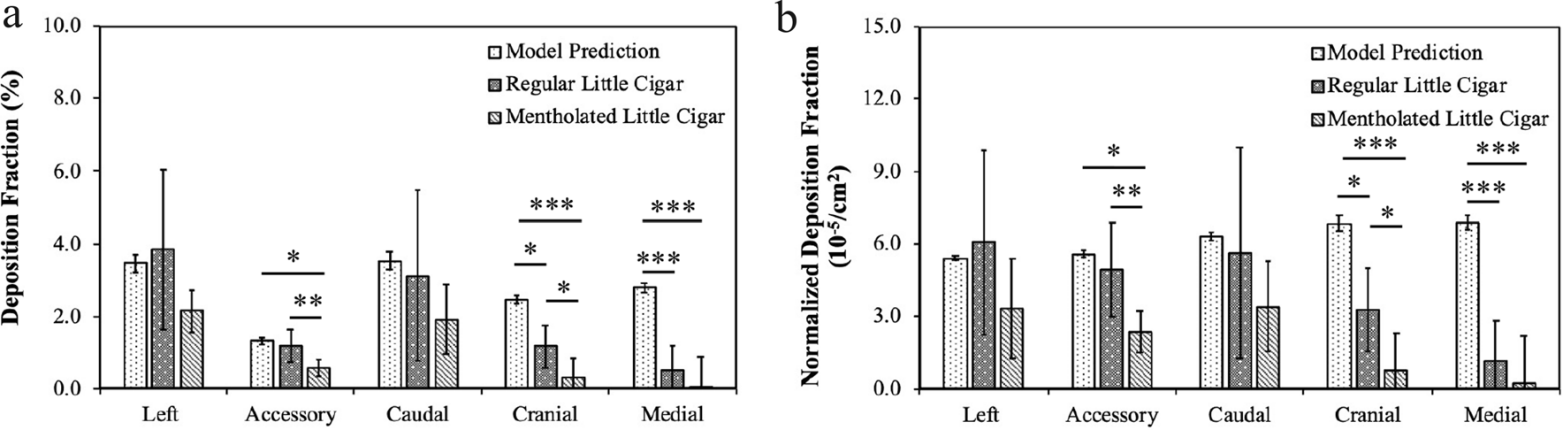
Comparison on the **a** deposition fraction and **b** normalized deposition fraction between MPPD model prediction and animal study results. Bars for the model predictions showed the mean ± standard deviation of male and female rats estimations. Model predictions were identical for regular and mentholated little cigars because they had a similar particle size distribution. Bars for animal study represent the mean ± standard error for the merged data from both male and female rats (n = 10). Kruskal–Wallis test, with a post-hoc Dunn’s test was conducted to compare the significance between the animal study and model prediction results. * indicates *P* < 0.05, ** indicates *P* < 0.01, and *** indicates *P* < 0.001

The MPPD model predicted only a minor difference in the lobe-specific deposition fraction and normalized deposition fraction between male and female rats. Thus, sex-averaged model outcomes were calculated for comparison with the results from animal studies. As illustrated in Fig. 6, the model projected a heterogeneous deposition fraction across the lobes. Specifically, the left and caudal lobes were predicted to have significantly greater particle deposition than the accessory lobe. The model demonstrated strong performance in predicting particle deposition in the left, accessory, and caudal lobes for regular little cigar smoke. However, significant discrepancies were noticed between the model and animal study results for the cranial and medial lobes, resulting in overestimations by several fold. As the regular and mentholated little cigars exhibited very similar particle size distributions (Additional file 1: Table S4), the MPPD model results were identical for the two products. Thus, the bars that represent model predictions indicated the modeling results for both regular and mentholated little cigars. In contrast, the animal studies showed that mentholated little cigars had an overall lower deposition fraction, which led to an even more pronounced overestimation from the MPPD models across all lobes.

Interestingly, the model predicted a homogeneous pattern among lobes for the normalized deposition fraction, mirroring observations in male rats. Variations among the five lobes were minimal (less than 25%) with the medial lobe predicted to deposit the highest number of particles per unit area. The experimental results from both sexes that were exposed to the same little cigar product were combined and plotted. As previously discussed, the animal study showed that male and female rats demonstrated different patterns in the normalized deposition fraction. Despite the potential for obfuscation of these differences by merging the data, it remained clear that the left and caudal lobes had a higher deposition per unit area than the cranial and medial lobes for both little cigar products. This pattern contrasts with the model prediction and suggests a need for optimization of the model to enhance its accuracy, especially with respect to the accessory, cranial, and medial lobes.

## Discussion

The goals of this study include understanding the deposition of little cigar smoke particle in rat lungs, exploring the distribution pattern of these particles across lobes, and comparing experimental results with model predictions. To the best of our knowledge, this is the first study to measure lung deposition of particles from this emerging tobacco product in rat models. Our results indicate that approximately 10% and 5% of the insoluble and soluble particulate constituents in the inhaled regular and mentholated little cigar smoke were deposited in the lobes of Sprague-Dawley rats, respectively. A previous study investigated the deposition of 1R3 research cigarettes particles in 7-month Fischer 344/N rats at higher exposure concentrations [34], which found a greater overall deposition fraction at 12.4–15.9%. The difference can be mainly attributed to the larger particle size (median mass aerodynamic diameter between 0.4 and 0.5 µm) and higher mean body weight in the cited study. Additionally, the difference may also be due to differences in smoke constituents [21, 35–37], such as higher levels of carbonyls and nicotine per gram of product, in little cigars compared to research cigarettes. These variations and the resulting differences in hygroscopic growth in the airways between research cigarettes and little cigar smoke are known to affect particle deposition in lungs [7, 38].

Several studies have previously explored lobe-specific particle deposition of research cigarettes in rats [34, 39]. Their results suggested a higher deposition fraction in the left and caudal lobes, as these two lobes exhibited the highest levels of radioactive particles. This is consistent with our findings. However, once the deposition fraction was normalized to the weight of the lobe in these prior studies, the difference was no longer significant, diverging from the heterogeneous pattern observed with little cigar smoke particles in our study. It is important to note that in the previous studies, particle deposition was normalized to the weight of wet lobe tissues rather than airway surface areas, as we have done in our study. During the necropsy and the sample collection process, various amount of blood in lobe tissues are impossible to separate from the tissues, introducing significant uncertainties to the calculated normalized deposition fractions. Thus, we believe airway surface areas of individual lobes, estimated based on rat mean body weight, offers a more accurate way to account for the size of lobes. Other recent modeling studies normalized to surface area as we did to calculate the deposition area density [40]. We further normalized the particle deposition fraction based on lobar volume (Additional file 1: Fig. S1) to determine if there would be any variation in the results. The deposition patterns appeared to be similar when comparing surface area and lobar volume normalization. When we extracted data from the study above employing research cigarettes and normalized the deposition fraction to airway surface areas, there appeared to be no significant difference in the normalized deposition fraction among lobes. The left and caudal lobes were even lower than other lobes, contradicting our animal study results. Clearly, caution should be used when interpreting and comparing results from studies that have applied different normalization approaches.

Interestingly, we observed a sex effect on distribution of little cigar smoke particles. A higher deposition fraction in the left and caudal lobes for both sexes suggested that larger amounts of smoke particles deposited in these two lobes compared to other regions. For male rats, this was primarily due to the greater airway surface areas of these two lobes, while the number of particles deposited per unit area remained relatively consistent across different lobes, except for the medial lobe. In contrast, both the normalized deposition fraction and airway surface areas were greater in the left and caudal lobes of female rats, leading to a more substantial difference among lobes. Differences in regional deposition of inhaled particles between male and female human subjects have been reported previously [41]. The reasons behind the variation remain unclear. Although the size of lung is consistently smaller in female rats compared to males, the difference is proportional across lobes, suggesting the size disparity may not be the primary reason for the disproportional particle distribution. It could instead be attributed to differences in adjustments to breathing patterns when exposed to high concentrations of smoke particles or due to anatomical difference in their lungs [42].

Menthol flavoring, which triggers the TRPM8 receptors, results in deeper inhalation and reduces the sensation of respiratory discomfort associated with loaded breathing [43–45]. As a result, smokers are more likely to hold the smoke and breath deeper [46], which in theory would lead to a higher particle deposition [47]. Additionally, study has reported a reduction in ciliary beating frequency following exposure to menthol-containing tobacco products, which impairs mucociliary clearance of deposited particle in airway [48, 49]. Based on this, we had hypothesized that the overall deposition fraction would be higher with mentholated little cigars than the regular little cigars. Contrary to our expectations, our results demonstrated the opposite. The overall deposition fraction of mentholated smoke particles in the five lobes combined, as well as in each individual lobe, was lower compared to regular little cigars. Despite the lower particle deposition fraction, the variations among lobes were more pronounced in both male and female rats (Fig. 5). These findings suggest the presence of unknown factors that influence particle deposition and distribution among lobes. Previous studies have shown that rat tidal volume, breath frequency, and minute volume significantly reduce during tobacco smoke exposure [39]. The dynamics become more complicated with the addition of menthol flavoring in little cigars, as they can alter the rats’ breathing pattern [46, 47]. Future research focusing on measuring rat breathing parameters during the exposure experiment and collecting data on the actual lung structure will be essential in answering these questions.

Given the vast number of tobacco products on the market, it is impractical to conduct animal experiments and investigate the particle deposition pattern for each one of them. Modeling serves as a potent tool for predicting tobacco smoke deposition in lungs. Results from these simulations are frequently used to inform policymakers and guide regulations. Hence, it is critical to ensure the accuracy of model simulation results. The uncertainties and limitations inherent in the measurements contribute to some of the observed discrepancy between the model predictions and experimental results. Respiratory breathing parameters are critical since they can significantly alter particle deposition yet were not characterized in this study. The aerosol density was estimated using data from research cigarettes, and this may vary slightly from that of little cigar smoke. Moreover, smoke particle chemical composition was not fully characterized. The differences also underscore the need for improvement in the current MPPD model, particularly with respect to predicting little cigar smoke deposition and providing lobe-by-lobe resolution. It is worth mentioning that the MPPD model version that was utilized in this study was not designed for tobacco smoke, which may partly explain the overestimations illustrated in Fig. 6. A few papers from the model’s developers suggest that the MPPD model has been adapted for applications related to tobacco smoke. A comparison between the experimental measurements and model simulations should be revisited once the new version of the model is released. Data from this study can be used by other model developers to optimize their models, such as symmetric single-path model [50], asymmetric multiple-path particle deposition (MPPD) [51], and asymmetric five-lobe stochastic lung model [52]. The ultimate goal is to improve these models so that the results from this rat study can translate to predict little cigar smoke deposition in human lungs.

## Conclusions

In summary, we measured the deposition of little cigar smoke particles in the lungs of Sprague-Dawley rats to investigate their spatial distribution across lobes. Our findings revealed that regular little cigars deposited more particles in the lobes of rat lungs compared to mentholated little cigars. The results demonstrated a heterogenous deposition pattern, with higher particle deposition observed in the left and caudal lobes, especially with the mentholated little cigars. Additionally, we identified disparities between our measurements and the MPPD model predictions. This discrepancy highlights the need to enhance the accuracy of models before extrapolating animal study results to human lung deposition. Overall, our study provides valuable insights for estimating the dose of little cigars during smoking for toxicity research.

### Supplementary Information

**Additional file 1**. Supplementary information.

## Data Availability

All relevant data are included in the manuscript and supporting information, and available from the authors upon request.

## References

[1] World Health Organization (2017). WHO Report on the Global Tobacco Epidemic, 2017: monitoring tobacco use and prevention policies.

[2] Dockery DW, Pope CA, Xu X, Spengler JD, Ware JH, Fay ME, Ferris BG, Speizer FE (1993). An association between air pollution and mortality in six US cities. N Engl J Med.

[3] Gasparotto J, Da Boit Martinello K (2021). Coal as an energy source and its impacts on human health. Energy Geosci.

[4] Jabbal S, Poli G, Lipworth B (2017). Does size really matter? Relationship of particle size to lung deposition and exhaled fraction. J Allergy Clin Immunol.

[5] Heyder J (2004). Deposition of inhaled particles in the human respiratory tract and consequences for regional targeting in respiratory drug delivery. Proc Am Thorac Soc.

[6] Morrow PE (1986). Factors determining hygroscopic aerosol deposition in airways. Physiol Rev.

[7] Varghese SK, Gangamma S (2006). Particle deposition in human respiratory tract: effect of water-soluble fraction. Aerosol Air Qual Res.

[8] Brand P, Friemel I, Meyer T, Schulz H, Heyder J, Häuβinger K (2000). Total deposition of therapeutic particles during spontaneous and controlled inhalations. J Pharm Sci.

[9] Abdollahi H, Babamiri A, Ahookhosh K, Farnoud A, Nabaei M. Effects of inhalation flow rate on particle deposition and flow structure in a model of tracheobronchial airway. *IEEE* **2021**, 101–106. 10.1109/ICBME54433.2021.9750358.

[10] Thakur AK, Kaundle B, Singh I. Mucoadhesive drug delivery systems in respiratory diseases. In Targeting chronic inflammatory lung diseases using advanced drug delivery systems. Elsevier; 2020. Pp. 475–491. 10.1016/B978-0-12-820658-4.00022-4

[11] Darquenne C (2020). Deposition mechanisms. J Aerosol Med Pulm Drug Deliv.

[12] Sosnowski TR (2011). Importance of airway geometry and respiratory parameters variability for particle deposition in the human respiratory tract. J Pharm Sci.

[13] Hussain M, Renate W-H, Werner H (2011). Effect of intersubject variability of extrathoracic morphometry, lung airways dimensions and respiratory parameters on particle deposition. J Thorac Dis.

[14] Brooks DR, Austin JHM, Heelan RT, Ginsberg MS, Shin V, Olson SH, Muscat JE, Stellman SD (2005). Influence of type of cigarette on peripheral versus central lung cancer. Cancer Epidemiol Biomarkers Prev.

[15] Park JD, Kim JK, Jo MS, Kim YH, Jeon KS, Lee JH, Faustman EM, Lee HK, Ahn K, Gulumian M, Oberdörster G, Yu IJ (2019). Lobar evenness of deposition/retention in rat lungs of inhaled silver nanoparticles: an approach for reducing animal use while maximizing endpoints. Part Fibre Toxicol.

[16] Lariscy JT (2019). Smoking-attributable mortality by cause of death in the united states: an indirect approach. SSM Popul Health.

[17] Food and Drug Administration, Department of Health and Human Services (HHS) (2022). Tobacco product standard for menthol in cigarettes. Fed Regist.

[18] Phan L, McNeel TS, Choi K (2021). Prevalence of current large cigar versus little cigar/cigarillo smoking among U.S. adults, 2018–2019. Prev. Med. Rep..

[19] Levy DT, Blackman K, Tauras J, Chaloupka FJ, Villanti AC, Niaura RS, Vallone DM, Abrams DB (2011). Quit attempts and quit rates among menthol and nonmenthol smokers in the United States. Am J Public Health.

[20] Schuster RM, Hertel AW, Mermelstein R (2013). Cigar, cigarillo, and little cigar use among current cigarette-smoking adolescents. Nicotine Tob Res.

[21] Reilly SM, Goel R, Bitzer Z, Elias RJ, Foulds J, Muscat J, Richie JP (2018). Little cigars, filtered cigars, and their carbonyl delivery relative to cigarettes. Nicotine Tob Res.

[22] Ghosh A, Abdelwahab SH, Reeber SL, Reidel B, Marklew AJ, Garrison AJ, Lee S, Dang H, Herring AH, Glish GL, Kesimer M, Tarran R (2017). Little cigars are more toxic than cigarettes and uniquely change the airway gene and protein expression. Sci Rep.

[23] King BA, Tynan MA, Dube SR, Arrazola R (2014). Flavored-little-cigar and flavored-cigarette use among U.S. middle and high school students. J Adolesc Health.

[24] Winkler-Heil R, Hofmann W (2016). Modeling particle deposition in the Balb/c mouse respiratory tract. Inhal Toxicol.

[25] Hofmann W (2000). The effect of heterogeneity of lung structure on particle deposition in the rat lung. Toxicol Sci.

[26] Sonnenberg AH, Herrmann J, Grinstaff MW, Suki B (2020). A Markov chain model of particle deposition in the lung. Sci Rep.

[27] Teague SV, Pinkerton KE, Goldsmith M, Gebremichael A, Chang S, Jenkins RA, Moneyhun JH (1994). Sidestream cigarette smoke generation and exposure system for environmental tobacco smoke studies. Inhal Toxicol.

[28] Anderson DS, Patchin ES, Silva RM, Uyeminami DL, Sharmah A, Guo T, Das GK, Brown JM, Shannahan J, Gordon T, Chen LC, Pinkerton KE, Van Winkle LS (2015). Influence of particle size on persistence and clearance of aerosolized silver nanoparticles in the rat lung. Toxicol Sci.

[29] Herner JD, Green PG, Kleeman MJ (2006). Measuring the trace elemental composition of size-resolved airborne particles. Environ Sci Technol.

[30] Johnson TJ, Olfert JS, Cabot R, Treacy C, Yurteri CU, Dickens C, McAughey J, Symonds JPR (2014). Steady-state measurement of the effective particle density of cigarette smoke. J Aerosol Sci.

[31] Silbaugh SA, Horvath SM (1982). Effect of acute carbon monoxide exposure on cardiopulmonary function of the awake rat. Toxicol Appl Pharmacol.

[32] Gu Z, Januszkiewicz AJ, Mayorga MA, Coleman GD, Morrissette CR (2005). Consequences of brief exposure to high concentrations of carbon monoxide in conscious rats. Inhal Toxicol.

[33] Lawrence D, Cadman B, Hoffman AC (2011). Sensory properties of menthol and smoking topography. Tob Induc Dis.

[34] Chen BT, Weber RE, Yeh HC, Lundgren DL, Snipes MB, Mauderly JL (1989). Deposition of cigarette smoke particles in the rat. Fundam Appl Toxicol.

[35] Hoffmann D, Wynder EL (1972). Smoke of cigarettes and little cigars: an analytical comparison. Science.

[36] Pickworth WB, Rosenberry ZR, Yi D, Pitts EN, Lord-Adem W, Koszowski B (2018). Cigarillo and little cigar mainstream smoke constituents from replicated human smoking. Chem Res Toxicol.

[37] Marusich JA, Wiley JL, Silinski MAR, Thomas BF, Meredith SE, Gahl RF, Jackson KJ (2019). Comparison of cigarette, little cigar, and waterpipe tobacco smoke condensate and e-cigarette aerosol condensate in a self-administration model. Behav Brain Res.

[38] Ching J, Kajino M (2018). Aerosol mixing state matters for particles deposition in human respiratory system. Sci Rep.

[39] Kendrick J (1976). Tobacco smoke inhalation studies in rats. Toxicol Appl Pharmacol.

[40] Winkler-Heil R, Hussain M, Hofmann W (2021). Predictions of inter- and intra-lobar deposition patterns of inhaled particles in a five-lobe lung model. Inhal Toxicol.

[41] Kim CS, Hu SC (1998). Regional deposition of inhaled particles in human lungs: comparison between men and women. J Appl Physiol.

[42] Zeman KL, Bennett WD (2009). Measuring alveolar dimensions at total lung capacity by aerosol-derived airway morphometry. J Aerosol Med.

[43] Klausner K (2011). Menthol cigarettes and smoking initiation: a tobacco industry perspective. Tob Control.

[44] Willis DN, Liu B, Ha MA, Jordt S, Morris JB (2011). Menthol attenuates respiratory irritation responses to multiple cigarette smoke irritants. FASEB J.

[45] Nishino T, Tagaito Y, Sakurai Y (1997). Nasal inhalation of *l* -Menthol reduces respiratory discomfort associated with loaded breathing. Am J Respir Crit Care Med.

[46] Watson CV, Richter P, De Castro BR, Sosnoff C, Potts J, Clark P, McCraw J, Yan X, Chambers D, Watson C (2017). Smoking behavior and exposure: results of a menthol cigarette cross-over study. Am J Health Behav.

[47] Darquenne C (2012). Aerosol deposition in health and disease. J Aerosol Med Pulm Drug Deliv.

[48] Baumlin N, Silswal N, Dennis JS, Niloy AJ, Kim MD, Salathe M (2023). Nebulized menthol impairs mucociliary clearance via TRPM8 and MUC5AC/MUC5B in primary airway epithelial cells. Int J Mol Sci.

[49] Herbert J, Kelty JS, Laskin JD, Laskin DL, Gow AJ (2023). Menthol flavoring in E-cigarette condensate causes pulmonary dysfunction and cytotoxicity in precision cut lung slices. Am J Physiol-Lung Cell Mol Physiol.

[50] Yeh H-C, Schum’ GM, Biomedical L, Box PO (1980). Models of human lung airways and their application to inhaled particle deposition. Bull Math Biol.

[51] Asgharian B, Hofmann W, Bergmann R (2001). Particle deposition in a multiple-path model of the human lung. Aerosol Sci Technol.

[52] Koblinger L, Hofmann W (1990). Monte Carlo modeling of aerosol deposition in human lungs. Part I: Simulation of particle transport in a stochastic lung structure. J Aerosol Sci.

