# Supplementary material for: Heterogeneous deposition of regular and mentholated little cigar smoke in the lungs of Sprague-Dawley rats
Source: Part Fibre Toxicol. 2023 Nov 6;20:42. doi: 10.1186/s12989-023-00554-6 (PMC10626780; doi:10.1186/s12989-023-00554-6)
Supplement: Supplementary file 1 — Additional file 1. Supplementary information. [file 12989_2023_554_MOESM1_ESM.docx]

# **Supporting Information**

# **Title: Heterogeneous Deposition of Regular and Mentholated Little Cigar Smoke in the Lungs of Sprague-Dawley Rats**

9 Pages, 1 Texts, 4 Tables, 1 Figure

Kaisen Lin^1,2 *^, Christopher Wallis^1^, Emily M. Wong^1,3^, Patricia Edwards^4^, Austin Cole^5^, Laura Van Winkle^3,4^, and Anthony S. Wexler^1,6,7,8^

^1^ Air Quality Research Center, University of California, Davis, Davis, CA 95616

^2^ Department of Civil and Environmental Engineering, Michigan State University, East Lansing, MI 48824

^3^ Department of Anatomy, Physiology and Cell Biology, University of California, Davis, Davis, CA 95616

^4^ Center for Health and the Environment, University of California, Davis, Davis, CA 95616

^5^ UC Davis Interdisciplinary Center for Plasma Mass Spectrometry, University of California, Davis, Davis, CA 95616

^6^ Department of Mechanical and Aerospace Engineering, University of California, Davis, Davis, CA 95616

^7^ Department of Civil and Environmental Engineering, University of California, Davis, Davis, CA 95616

^8^ Department of Land, Air, and Water Resources, University of California, Davis, Davis, CA 95616

**Supporting Information**

Text 1: ICP-MS analysis method.

Table S1: Filter extraction efficiency.

Table S2: Rat airway surface areas.

Table S3: Rat lobar volume.

Table S4: Little cigar smoke particle size.

Figure S1: Lobar volume-normalized deposition fraction.

**Analysis on Air Filter Extracts and Lung Sample Digests**

The Agilent 8900 ICP-MS Triple Quad instrument (Agilent Technologies, 5301 Stevens Creek Blvd., Santa Clara, CA 95051) equipped with an inert PFA sample introduction kit, Pt cones, and a brass baseplate was tuned and calibrated prior to analysis of the prepared samples and operated in MS/MS Nogas, H_2_, He, and O_2_ modes using a 3-point peak pattern with 3 replicates per injection and 50 sweeps per replicate. H_2_, He, or O_2_ was used in the collision/reaction cell during the measurements to reduce polyatomic interferences or mass-shift an element of interest away from interfering masses. Digested samples and digestion batch Quality Control (QC) standards, external calibration standards, check standards, and blanks were sampled using an Agilent SPS 4 Autosampler equipped with a 0.5mm ID sample probe, mixed at ~18:1 ratio with a custom internal standard solution using a mixing tee, then introduced into the ICP-MS via peristaltic pump at 0.10 rps using a 200 µL/min PFA concentric nebulizer to produce an aerosol in a 2°C temperature-controlled PFA double pass Scott-type spray chamber leading to a 1550 W plasma via an inert sapphire injector.

External calibration standards were diluted from a custom Inorganic Ventures (Inorganic Ventures, Inc., 300 Technology Drive, Christiansburg, VA 24073) multi-element standard mix. A custom internal standard solution with Sc, Ge, Y, In, and Bi was prepared from Inorganic Ventures single element standards. Concentrated TraceMetals Grade HNO_3_ and HCl, and 18.2 MΩ/cm water MilliQ Water, was used to prepare external calibration standards, check standards, and samples at '33.3% conc. HNO_3_/11.1% conc. HCl’ in 15mL centrifuge tubes, and the internal standard solution in a 50mL centrifuge tube (Thermo Fisher Scientific, 168 Third Ave., Waltham, MA 02451). NIST1643f - Trace Elements in Water (National Institute of Standards and Technology, 100 Bureau Drive, Gaithersburg, MD 20899), a single-element Ti Inorganic Ventures standard, and a blank were analyzed initially for independent source QC calibration and blank verification. Custom multi-element Inorganic Ventures standard mix dilutions and a blank were analyzed before and after no more than 10 digested samples or digestion batch QC injections to monitor instrument performance and provide continuing calibration and blank verification. The raw data was processed using MassHunter ICP-MS software (G7201C, Version C.01.06, Agilent) and a correction equation was used sum the 206, 207, and 208Pb isotope signals for Pb reporting.

**Table S1.** The extraction efficiency of little cigar smoke particles from PTFE filters with 75% acetone and 25% 1N nitric acid.

| **Exposure Experiment** | **Mass Extracted (mg)** | **Mass Collected (mg)** | **Extraction Efficiency (%)** |
| --- | --- | --- | --- |
| Mentholated_Female | 3.09 | 3.60 | 85.91 |
| Mentholated_Male | 1.72 | 1.97 | 87.20 |
| Regular_Male | 1.72 | 1.87 | 91.68 |
| Regular_Female | 1.93 | 2.26 | 85.38 |

**Table S2.** The airway surface areas by lobes projected by MPPD model for male and female Sprague-Dawley rats based on body weight.

| **Lobes** | **Female Rats (cm^2^)** | **Male Rats (cm^2^)** |
| --- | --- | --- |
| Left | 595.58 | 680.31 |
| Accessory | 218.11 | 249.14 |
| Caudal | 522.90 | 597.28 |
| Cranial | 333.55 | 381.01 |
| Medial | 377.31 | 430.95 |

**Table S3.** The lobar volume projected by MPPD model for male and female Sprague-Dawley rats based on body weight.

| **Lobes** | **Female Rats (cm^3^)** | **Male Rats (cm^3^)** |
| --- | --- | --- |
| Left | 0.96 | 1.22 |
| Accessory | 0.35 | 0.44 |
| Caudal | 0.83 | 1.07 |
| Cranial | 0.53 | 0.67 |
| Medial | 0.59 | 0.76 |

**Table S4.** The primary peaks, secondary peaks, and geometric standard deviations of particle size distribution from regular and mentholated little cigar.

| **Exposure** | **Primary Peak (nm)** | **Secondary Peak (nm)** | **Geometric Standard Deviation** |
| --- | --- | --- | --- |
| Mentholated-Female | 358.7 | 126.3 | 1.78 |
| Mentholated-Male | 358.7 | 145.9 | 1.75 |
| Regular-Male | 371.8 | 121.9 | 1.84 |
| Regular-Female | 358.7 | 140.7 | 1.77 |

**
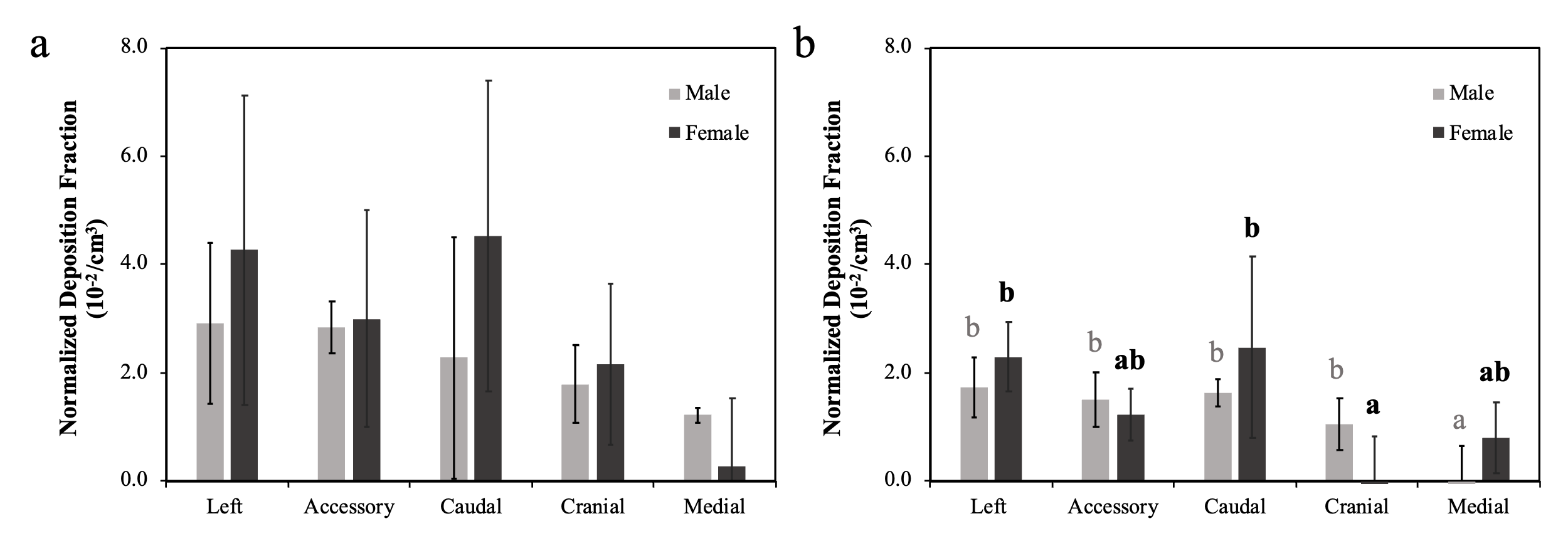
**

**Figure S1**. Lobar volume-normalized deposition fraction of (a) regular and (b) mentholated little cigar smoke in male and female rat’s lungs. Bars represent mean ± standard error for each lobe (n=5). Bars with the different letters indicate significant difference (P < 0.05) between lobes within the same sex of rats according to one-way ANOVA with the Tukey’s HSD test. Statistical analyses on male and female rats’ data were conducted separately and thus the annotated letters should be interpreted separately. There were no statistical differences in the normalized deposition fraction across the lobes for male and female rats exposed to regular little cigars. As a result, panel (a) does not contain any letter annotations.
